# Gender Beneath the Skull: Agency, Trauma and Persisting Stereotypes in Neuroepigenetics

**DOI:** 10.3389/fnhum.2021.667896

**Published:** 2021-06-15

**Authors:** Elsher Lawson-Boyd, Maurizio Meloni

**Affiliations:** Alfred Deakin Institute for Citizenship and Globalisation, Deakin University, Burwood, VIC, Australia

**Keywords:** neuroepigenetics, gender, trauma, plasticity, interdisciplinarity, family violence, qualitative research, neuroscience

## Abstract

Epigenetics stands in a complex relationship to issues of sex and gender. As a scientific field, it has been heavily criticized for disproportionately targeting the maternal body and reproducing deterministic views of biological sex (Kenney and Müller, 2017; Lappé, 2018; Richardson et al., 2014). And yet, it also represents the culmination of a long tradition of engaging with developmental biology as a feminist cause, because of the dispersal of the supposed ‘master code’ of DNA among wider cellular, organismic and ecological contexts (Keller, 1988). In this paper, we explore a number of tensions at the intersection of sex, gender and trauma that are playing out in the emerging area of neuroepigenetics - a relatively new subfield of epigenetics specifically interested in environment-brain relations through epigenetic modifications in neurons. Using qualitative interviews with leading scientists, we explore how trauma is conceptualized in neuroepigenetics, paying attention to its gendered dimensions. We address a number of concerns raised by feminist STS researchers in regard to epigenetics, and illustrate why we believe close engagement with neuroepigenetic claims, and neuroepigenetic researchers themselves, is a crucial step for social scientists interested in questions of embodiment and trauma. We argue this for three reasons: (1) Neuroepigenetic studies are recognizing the agential capacities of biological materials such as genes, neurotransmitters and methyl groups, and how they influence memory formation; (2) Neuroepigenetic conceptions of trauma are yet to be robustly coupled with social and anthropological theories of violence (Eliot, 2021; Nelson, 2021; Walby, 2013); (3) In spite of the gendered assumptions we find in neuroepigenetics, there are fruitful spaces – through collaboration – to be conceptualizing gender beyond culture-biology and nature-nurture binaries (Lock and Nguyen, 2010). To borrow Gravlee’s (2009: 51) phrase, we find reason for social scientists to consider how gender is not only constructed, but how it may “become biology” via epigenetic and other biological pathways. Ultimately, we argue that a robust epigenetic methodology is one which values the integrity of expertise outside its own field, and can have an open, not empty mind to cross-disciplinary dialogue.

## Introduction: Biology Beyond the Genome

In the wake of the Human Genome Project, molecular biology has undergone some fascinating changes. In particular, common understandings pertaining to the simple genetic determination of phenotypes is being challenged by advances in postgenomic sciences. What has been called “missing heritability” – the elusive correlation between genetic variants and common traits or complex diseases – not only exemplifies the lack of explanatory power of gene-centric explanations (Maher, 2008; Pennisi, 2012; Lock, 2015) but has increasingly pushed scientists to look at the wider architectural complexity surrounding gene expression, from cell to society, to account for environmentally sensitive variations in bodies, health and disease (Keller, 2011, 2014; Landecker, 2011; Niewohner, 2011). Rather than genetic mutations being unresponsive to the environment during the lifespan, postgenomic thinking explores how the environment comes into the body and modulates the genome in relatively short time frames (Keller, 2010, 2015; Charney, 2012; Landecker and Panofsky, 2013; Moore, 2015; Warin and Martin, 2018).

This postgenomic shift (Griffiths and Stotz, 2013; Richardson and Stevens, 2015; Meloni, 2016, 2019; Baedke, 2018) parallels the growing appreciation of experience-dependent plasticity in the human brain throughout life that “has drawn attention to the crucial role that the outside world—the lives we live, the jobs we do, the sports we play” continuously have on brain functioning and the nervous system (May, 2011; Chang, 2014). As neuroscientist Gina Rippon (2019: 235) notes:

It’s no longer a question of our brains being a product of either nature or nurture but realizing how entangled the “nature” of our brains is with the brain-changing “nurture” provided by our life experiences.

A similar move toward an entanglement of nature, nurture and plasticity of genomic functioning is represented by the expanding field of epigenetics: the study of mitotically (cell division) or meiotically (sex cell division) heritable changes in gene function that cannot be explained by changes in genetic sequence. In epigenetic language, environmental “signals” and “exposures” are said to alter the configuration of epigenetic markers, such as methylation (i.e., the addition or subtraction of methyl groups), non-coding RNAs and histone acetylation. Through these contemporary reconfigurations, genes are understood less as growing and operating on their own. Instead, they are considered part of a complex biochemical assemblage that - by virtue of its material constitution - allows for change and transformation in response to the surroundings of a cell, organ and organism in the broadest (and perhaps vaguest) sense. Some epigenetic studies (primarily using animal models) are also suggesting that epigenetic modifications can be carried through the germ line, and thus can be inherited across generations (Jablonka, 2013; Sharma, 2013).

Based on some of the cross-disciplinary commentary of epigenetics, especially feminist Science and Technology Studies (STS), it is clear that the field of epigenetics stands in a complex relationship to issues of sex and gender. As a scientific field, it has been heavily criticized for disproportionately targeting the maternal body and reproducing deterministic views of biological sex (Richardson et al., 2014; Kenney and Müller, 2017; Lappé, 2018). And yet, it also represents a long tradition of engaging with developmental biology as a feminist cause, because of the dispersal of the supposed “master code” of DNA among wider cellular, organismic and ecological contexts (Haraway, 1976; Keller, 1997, 2002). Moreover, it is from epigenetics that some less known but fascinating studies on paternal exposures (nutrition, stress, and smoking) have recently arisen, complicating the usual focus on maternal blame when it comes to “pathologizing” effects on their offspring (Rando, 2012, 2016; Rodgers et al., 2013; Soubry et al., 2013; Gapp et al., 2014, 2016; Soubry, 2015; Andaloussi et al., 2019; Le Blévec et al., 2020). This reorientation is part of a wider evolutionary rethinking about the role of paternal care (or biparental care) in mammals, which has emerged in these last few years (Pilakouta et al., 2018). Although there is clearly a shift in interest toward paternal epigenetic transmission - a shift which has been welcomed by many in the field – the conceptual discussion about gender - as lived, multiple and complex - is lacking. This is especially clear in studies exploring the epigenetic impact, imprint and transmission of trauma and stress.

In this paper, we explore a number of tensions at the intersection of sex, gender and trauma that are playing out in the emerging area of neuroepigenetics - a relatively new subfield of epigenetics specifically interested in environment-brain relations through epigenetic modifications in neurons, which may subsequently affect their function, lifespan and capacity to retain memories. Although still in its infancy, neuroepigenetics is particularly salient as it arises at the crossroads of two trends that have attracted much attention over the last few decades: firstly, the biological embedding of social experience, meaning the process whereby life experiences produce “lasting changes in the function of a biological system with consequences for development, behavior, and health” (Aristizabal et al., 2020). Secondly, what sociologist Nikolas Rose and colleagues have recently termed “neuroecosociality,” an integration of biological and social understandings that builds upon emerging findings from neuroscience to find mechanistic pathways that explain trajectories of well-being and disease (Rose et al., 2021).

In a series of qualitative interviews^1^ with leading scientists carrying out research on epigenetic effects in the brain, we explore how trauma is conceptualized in neuroepigenetics, paying particular attention to its gendered dimensions. In so doing we address a number of concerns raised by feminist STS researchers in regard to epigenetics, sex and gender and illustrate why we believe close engagement with neuroepigenetic claims, and neuroepigenetic researchers themselves, is a crucial step for social scientists interested in questions of embodiment and trauma. We argue this for three reasons. Firstly, neuroepigenetic studies are recognizing the agential and reactive capacities of biological materials such as genes, methyl groups and particular sections of the brain, and how they affect the body’s stress response system and memory (Reul, 2014). Secondly, neuroepigenetic conceptions of trauma are yet to be robustly coupled with social and anthropological theories of violence (Walby, 2013; Eliot, 2021; Nelson, 2021). Many scientists interviewed for this study are engaging in questions about embodied and inherited trauma, yet in interviews as well as in peer-reviewed journal articles, the issue of types of violence has been scarcely mentioned. This may be in part attributed to specialization between disciplines institutionally, as well as lack of cross-disciplinary dialogue. For example, neuroscientist Thomas Lai^2^ (Melbourne) in our interview expressed frustration with a supposedly lack of productive cross-disciplinary conversation:

TL: It struck me that […] people aren’t really talking to other people, and there’s a danger in that, because you are so single minded and focused on your own pursuits that it’s very dangerous, if you’re going to ignore the other fields as well. You shouldn’t be excluding them; you should be incorporating them into your work.

In response to researchers like Thomas, we use this paper to raise a very simple but vital methodological step available to all parties: cross-disciplinary dialogue.

Thirdly, in spite of the problematic assumptions we find in neuroepigenetics regarding gender, there are also fruitful spaces – through collaboration – to be conceptualizing gender beyond culture-biology and nature-nurture binaries (Lock and Nguyen, 2010; Lock and Pálsson, 2016). It is important to note that the third point is more peripheral as far as this paper’s scope goes, but we raise it here nonetheless because our exploration of gender and trauma in a neuroepigenetic context has inevitably led to questions about the relations between exposures and “material bodily difference” (Guthman, 2014). Bodily difference, as a construct informed by postgenomic ideas, gestures less to a body pulled between the boundaries of nature and nurture, and more toward reactive, relational conceptions of human life and embodiment (Lock and Pálsson, 2016). To borrow (Gravlee’s, 2009: 51) phrase, we find reason for social scientists to consider how gender is not only constructed, but how it may “become biology” via epigenetic and other biological pathways. Thus, we argue that there is a need for feminist STS to be considering “biosocial differentiation” (i.e., the ways in which bodies and bodily substrates are modified relative to history, politics, economics and socialities in multiple time scales), and how modes of gendered violence play a role in this differentiation (Lock and Nguyen, 2010). As it has been shown in the case of racism and food justice, epigenetics lends itself to biosocial conceptualizations of difference as simultaneously cause and consequence of social injustices, without reducing them to matters of genetics or culture alone (Guthman, 2014).

Beyond this paper, and with insights from the postgenomic sciences, we see empirical opportunities for social scientists to consider gender – as well as gender inequality and violence – as a biologically absorbable, differentiating and transmittable agent (Roy, 2016; Cortés et al., 2019). The recent history of feminist theory can be characterized by overt concern of biological reductionism, determinism or evolutionary explanations (often driven by genetics), of sexual differences, as a way to naturalize (and hence justify) existing social and cultural inequalities and behavioral or psychological dimorphism between women and men (Fausto-Sterling, 1993, 2012; Fine, 2010; Richardson, 2013; Joel and Vikhanski, 2019; Mikkola, 2019). However, akin to our interlocutors from feminist neuroscience (Roy, 2016; Fine et al., 2017; Rippon, 2020), critical neuroscience (Choudhury and Slaby, 2011) and feminist STS (Haraway, 1988; Wilson, 2004), we believe that multi-disciplinary analyses of biosocial difference to be a progressive epistemological step, and will no doubt raise questions about the ways in which social justice movements conceptualize the body.

In these encounters, we anticipate that a “critical friendship” between neuroepigenetics, feminist STS and medical anthropology – however precarious a process this may be – will not run counter to emancipatory ends, but support them (Rose and Abi-Rached, 2013; Fitzgerald and Callard, 2015; Roy, 2016). Although we do not attribute any inherent emancipatory meaning by itself to epigenetics (Mansfield and Guthman, 2015), the peculiar hybrid nature of knowledge production in epigenetics, which constantly criss-cross the boundaries between the social and the biological, may prove a very fertile ground to put to test the feminist incorporation of molecular biology (or molecular feminisms) in order to generate differences “not through lack but rather through positive and productive senses” (Roy, 2018). There is potential for neuroepigenetics not simply to challenge the notion that biology and biological processes are naturally “essentializing or deterministic” (Roy, 2018: 5), but also to question how particular neurobiological “actants” – hormones, genes, synapses and neurotransmitters – play their part in the making of subjectivities (or phenotypes) that is uncleavable from social, political and geographical exposures (Richardson, 2017; Cortés et al., 2019).

We say actants rather than “substrates,” “bases,” or “underpinnings,” because we believe that there is scope within epigenetics to challenge naively foundationalist views of biology according to which, in Susan Oyama’s developmentalist critique, one can move from the social to the biological as going “‘down’ the layers (…) from effect to cause, from the provisional to the immutable, from the trivial to the profound (….)” (Oyama, 2000: 164–5; Meloni, 2014). Also, in the words of philosopher Samantha Frost (2016, 2020), there is potential in epigenetics to imbue matter with meaning and agency, breaking the supposed association of fleshiness “with the unintelligent and the imperceptive.” The *attentive body* that she sees emerging from a theoretically aware connection of epigenetic science and living experience, can be refashioned here as an “attentive brain,” where epigenetic marks of trauma are not just an inert sign established by blind mechanistic forces, but part of the wider “embodied responsiveness” of an organism-in-context that “is at once inhabited by the traces of its past and seeded with traces of its future” (Meloni and Testa, 2014: 15; Frost, 2020; see also Meloni and Reynolds, 2020).

Ultimately, we argue that a robust and feminist postgenomic methodology is one which values the integrity of expertise outside its own field, and can have an open, not empty mind to cross-disciplinary dialogue. We foreground this argument in this special issue primarily because it has come from neuroepigenetic scientists themselves, who are calling for cultural reorientation which can respectfully account for other modes of knowledge, other reference points, and genuine collaboration across disciplinary fields.

To make this point a more salient one, and indeed, to gesture to the notion that gender may indeed have neurobiological agency (Higgins, 2018), in section four we make reference to the concerning rates of family violence during the COVID-19 crisis in Australia (where we are both based). We hope this will also illustrate why epigenetic researchers need to be cautious of unknowingly re-hashing gendered stereotypes, as they have epistemological consequences for the ways in which trauma research is carried out. Our attention to a particularly invisible type of violence - which remains socially, culturally and politically widespread, yet necessarily provincial – acts as both a caution about making gender-based assumptions, and a rationale as to why neuroepigenetic conceptualizations of trauma at the very least need to be informed by empirical and theoretical studies of violence. In the vein of our epistemological commitment, and in a feminist STS tradition, we urge biological and social scientists to consider what allowances and restrictions their positioned perspective offers (Haraway, 1988). A point on our methodology: we assume and trust in first impressions (Schwartz, 2002); that what was said during interviews is to be taken as genuine, at “face value” and in good will.

## Neuroepigenetics: Neurobiological Change and Memory

Formally introduced by neurobiologist Jeremy Day (University of Alabama at Birmingham) and neuroscientist David Sweatt (Vanderbilt University), neuroepigenetics is foregrounded not only as reformulating “the fundamental existential question of nature versus nurture” but also as having the potential to sharpen current knowledge about the cognitive and psychic impacts of life experiences (Day and Sweatt, 2011; Kim et al., 2018; Coda and Gräff, 2020). Evoking the controversial theory of the “engram” - a (hypothetical) biophysical change in the brain that accounts for the material existence of memory (Josselyn et al., 2015: 201) - Day and Sweatt suggest that epigenetic mechanisms, such as DNA methylation, may be a window into the brain’s memory. The epigenome is said to be a crucial “missing link” between life experiences and gene expression, which in turn will influence the ways in which neuronal circuitry and brain structures develop. In regard to Post-Traumatic Stress Disorder, Day (University of Alabama at Birmingham, 2015)^3^ claims an existing correlation between neuro-epigenetic markers, memory and traumatic experience; by removing or altering epigenetic markers, the “negative” impacts of trauma may be manipulated and even possibly erased (Schmidt et al., 2013).

While neuroepigenetics has emerged only in recent years, the relationship between epigenetic changes and memory is far from being occasional or incidental. Firstly, as Day and Sweatt’s reference to the engram model evidences, epigenetic views of embedded experiences in the brain today resonate with influential late nineteenth and early twentieth century models of organic memory, although not necessarily adhering to the same neo-Lamarckian framework (Semon, 1921; Otis, 1994; Schacter, 2001; Szyf, 2014; Logan, 2015). Contemporary ideas of plasticity, brain receptiveness, experiential inscription and traces were a major part of these post-Darwinian debates that were later challenged by the rise of genetics (Chiapperino and Panese, 2019).

Secondly, epigenetics has opened new avenues in the last decade to wider research programs on synaptic plasticity and the neurobiology of memory (Landry et al., 2013) highlighting how epigenetic functioning and chromatin reshaping may underpin short and long-term memory processes, associative learning, and social cognition (Fagiolini et al., 2009; Ferrari et al., 2013; Post, 2016). By this, memory and indeed experience reflects biological differentiation. Epigenetic markers - or the epigenome - are said to act as the “the molecular memory” of by-gone stimuli, which allows a cell to “remember” past events that an organism has experienced (Bonasio et al., 2010: 612; see for a precursor Holliday, 1999). In the context of our article, we highlight in particular recent research on the importance of sex-specific epigenetic patterns in early life as a form of cellular memory that contributes to the establishment in adulthood of brain sex differences in animal models (McCarthy et al., 2017).

Thirdly, key epigenetic studies in animal models originate from or directly cut across neuroscience research which gravitate around topics of trauma, stress and their potential transmission across generations (which still remains a controversial argument) (Weaver et al., 2002, 2004; McGowan et al., 2011; Dias and Ressler, 2014). Since the late 1990s neuroscientists and molecular biologists have been fascinated with the brain’s capacity to be physically shaped by its social and material environment, particularly the maternal environment (Kim, 2021), especially during the earliest years of life (Francis et al., 1999; Coda and Gräff, 2020). During this time, the argument goes, neural cells are rapidly dividing as the body grows and thus the brain is perceivably more vulnerable, or in other words, more plastic (Fagiolini et al., 2009; Szyf, 2009). Not only are epigenetics considered mechanistically crucial for the shaping of neural pathways, but also for the ways in which somatic cells differentiate and perpetuate cellular phenotypes over time (Feinberg, 2007). Epigenetic changes in gene expression thus have emerged as an important mechanism that mediates the brain’s structural plasticity in periods of particular sensitivity (Cortés-Mendoza et al., 2013; Babenko et al., 2015).

Emerging from earlier research programs on the neuroepigenetics of memory (Zovkic et al., 2013a,b), the rate of neuroepigenetic studies on trauma, adversity and mental illness are growing fast, in some cases including claims about transgenerational inheritance or collective/historical trauma (Curry, 2019; see overview in Thayer et al., 2017; Yehuda and Lehrner, 2018; Dubois and Guaspare, 2020; Warin et al., 2020). For instance, a recently published edited book (Rutten, 2018) includes a selection of work addressing topics such as the transgenerational epigenetics of stress (Jawaid et al., 2018), neuroepigenetics of PTSD (Kim et al., 2018), as well as the central neuroepigenetic regulation of the hypothalamic-pituitary-adrenal-axis (Dick and Provencal, 2018; Montenegro et al., 2019). In addition, a *Frontiers* special issue on “Epigenetic Pathways to PTSD” featured thirteen articles from DNA methylation as biomarker in the detection of PTSD, to sex-specificity of stress responses (Roth, 2014).

Importantly, the studies included in the special issue suggest that genes have innate sensitivity and responsiveness which may have significant consequences for the ways in the nervous system – and in particular, stress response system – develops. As Roth (ibid., 3) explains:

Epigenetic mechanisms are a class of molecular mechanisms by which environmental influences, including stress, can interact with the genome to have long-term consequences for brain plasticity and behavior. As PTSD, by definition, requires exposure to a traumatic event, and because genes are exquisitely sensitive to stress and trauma, epigenetic alterations have received attention as possible contributors to the development and persistence of PTSD symptoms.

As two key scientists in the field, Michael Meaney (McGill, Montreal) and Rachel Yehuda (Mount Sinai Hospital, New York) observe in a co-authored chapter, this is not quite the same that noting the well-recognized “transient alterations in neural, endocrine or immunological signals that follow exposure to trauma”; unlike those “transient” variations, there is a stronger emphasis that “certain epigenetic markers can be chemically *stable* over extended periods of time and thus serve as the basis for an understanding of the *persistence* of PTSD symptoms” (2018: 293-294, our italics). Nonetheless, persistence does not mean fatalism, as in classical ‘faulty gene’ narratives: DNA methylation, while chemically stable, is *nevertheless reversible*, offering potential insights into future treatments for PTSD” (ibid.: our italics). We will explore in our interviews these tensions between passivity and agency, determination and reversibility, trauma and hope, that shape one of the key narratives of scientists in the field.

To sum up, if epigenetic research discursively blurs the line between body and milieu, allowing environments to “get under the skin” (McEwen, 2012), then neuroepigenetics looks at experiential *traces going under the skull*, and become *literally engraved* – for better or for worse – in the brain (Plazas-Mayorca and Vrana, 2011). Although the discussion we provide above will no doubt evoke suspicions for social scientists vis-a-vis the ways which neurobiology is materially and discursively responsive, beyond the potential hype and hope we find that neuroepigenetic ideations present an opportunity to frame the nervous system as a psychosomatic political site, where questions of gender, trauma and biosocial differentiation emerge. Based on neuroepigenetic ideas of neurological impressionability, as well as the nervous system’s vulnerability to “exposures” as a result, we urge social scientists to re-consider the agential capacities of flesh and what it might mean for analyses of violence. There are of course potential pitfalls in this kind of figuring, yet based on the advocacy expressed in range of literatures on bio-psycho-socialities (Blackman, 2016), feminist neuroscience (Roy, 2016), disability studies (Shakespeare, 2006; Goodley, 2011) as well as metabolism studies (Solomon, 2016) and food sovereignty (Guthman, 2014), neuroepigenetics lends itself well to an articulation of embodiment that may simultaneously decenter and situate the brain without reducing its material complexity (Roy, 2016).

If neurobiology *gets* and stays different through exposure, we ask here how much of it gets gendered too, by virtue of its material and relational constitution. And if it does, how can we incorporate a wider and sharper scope of social, political and biological agents into our analyses where matter and mattering are taken seriously “against the limits” of representational or constructionist paradigms (Grosz, 1994; Pitts-Taylor, 2016). We find this a pressing and timely task, not only as it points to generative possibilities for collaborative research, but also because of the ripple effects neuroepigenetic research may have in other nascent fields. From this brief review of current neuroepigenetic postulations, we find that the brain’s responsiveness to trauma is clearly at the frontier of this growing field, yet we also find a number of issues regarding trauma’s conceptual parameters, as well as the way in which types of violence are being neglected. In both the neuroepigenetic literature and our own interviews, the nuances of particular types of violence such as interpersonal, structural, collective and gender-based (Rutherford et al., 2007) are seldom addressed as agential. Moreover, definitions of stress and trauma are situated primarily in physiological, reproductive and neurological processes of the *sufferer*, which raises the question of how social and political agents can be given credence in this framing. This is a significant issue for the field, and we suggest that further contributions from feminist STS and medical anthropology are needed to address this. In the next section, we discuss some conceptions of trauma as described by leading neuroepigenetic researchers.

## Trauma’s Neuroepigenetic Agency/Legacy

Trauma, often taken for granted in both scientific and popular literature, is not a clearly defined object existing “there” in the world, nor is it a timeless category. Applying Allan Young’s classic analysis of PTSD, we can also say that trauma as a distinctive concept and independent disease is the relatively recent historical result of a number of narratives, technologies and epistemic practices “with which it is diagnosed, studied, treated, and represented by various interests, institutions, and moral arguments that mobilized these efforts and resources” (Young, 1995: 5). We see the growing centrality of ideas of trauma in epigenetics and neuropigenetics literature emerging at the intersection (or possibly culmination) of three important cultural trends that have taken place over the last two decades.

Firstly, there has been a massive expansion and cultural legitimation of ideas of traumatic victimhood in political, legal, and humanitarian contexts (Fassin and Rechtman, 2009) and more recently, in healthcare (Müller and Kenney, 2020). Secondly, there has been a shift from a psychodynamic view of trauma (i.e., trauma as the result of a conflict within the subject triggered by external conditions) to a *literalist* view of trauma as a “reality imprint in the brain (…) undistorted and uncontaminated by subjective meaning” (Leys, 2000: 7, see also Chapter 8). That is, to simplify, while a psychodynamic model emphasized unconscious conflicts internal to the subject as a source of trauma, the literal model highlights the pre-representational, veridical nature of trauma as literally engraved or etched in the brain beyond and before cognition (Leys, 2000, pp. 250 and ff.). Thirdly, emerging forms of “biolegitimacy” (Fassin, 2009) in which biologically validated knowledge about suffering is turned into a platform for political recognition. Described as “an historical testimony of colonial violence” (Warin et al., 2020: 4), epigenetics and other versions of bio-legitimized trauma are part of a growing trend where biological knowledge is recognized as possessing more authority than other forms of witnessing trauma (historical, narrative, phenomenological etc.).

In interviews with neuroepigenetic researchers, we explored how trauma is defined and recognized in their field. Although interviewees generally considered trauma elusive and subjective (i.e., it doesn’t appear to have a universal, clearly defined or standardized definition), they describe trauma as, in part, an objective neurological phenomenon innate to sufferers, and that can be better understood and treated once underlying biochemical mechanisms involved are identified. Trauma involves a plethora of chemical, neurological and cellular agents, thus rendering traumatic experiences neurologically affective in temporal, material and *sticky* ways. In other words, trauma – unlike stress, or far more than stress – stays under the skull and is particularly difficult, yet possible, to “fix.” For instance, when talking to Professor Alexander Berman, a veteran and leader in the field of epigenetics, he explained:

AB: You know what is stressful is subjective, but the only objective criteria we have is release of the stress hormone. So, if you define anything that releases stress hormone consistently it is stressful, and in fact it might vary between people right? But I think in the end it has to be mediated through some biochemical pathway, and the medical consequences will be how much this fires, so I will say that as a biochemist you know I will define it as a release of stress hormones and consistency of the release of stress hormones, but they could be released by anything.

Similarly, neuroepigenetic researcher Professor Lukas Birrer described trauma as “an extreme form of stress”; a little bit of stress is for the most part beneficial, he explained, but when “it becomes too much or prolonged, then this can lead to unhealthy consequences” for the body. Here, the discursive “consequences” of trauma (which we can assume to include psychiatric outcomes such as Post-Traumatic-Stress-Disorder etc.) are in part a result of temporally excessive amounts of stress hormone released in response to a significant (and vaguely defined) stressor.

The notion of consequence – be that medical, unhealthy or otherwise – begs a number of questions for epigenetic scientists regarding matters of ontology and scale: *what* characterizes respective neurological and epigenetic markings pertain to a past stressor, and what might be the negative effects of those markings for minds, bodies and collectives? Although the potential answers to these questions remain conceptually ambiguous and empirically contentious within the field, what we find to be consensus is the necessity to and relative difficulty of removing these markings.

In an interview with Adele Charlier, a leading neuroepigenetic researcher based in Western Europe, we asked what differentiates stress from trauma, to which she explained:

AC: Stress is usually defined biologically by the stress pathway. Or the release of stress hormones, due to something which happened to you which can be acute or chronic. Trauma is something very different, not very different, trauma includes some of the stress effect, but it has more to it…There is not enough knowledge to know exactly what epigenetic alterations can be repaired, because I don’t think there is a single answer to this. It depends on when and to what extent the epigenome has been interfered with, modified. You know, if it happens early in life, due to a chronic exposure, I think it’s quite intuitive to me, that it’s going to be embedded into the body and that may be difficult to. fix this, or normalize or correct it, because the system will have never been normal, in a way. It’s the same with psychiatric disorders. Children who are abused…who are exposed to trauma very early on, it just shapes their body, and then if something is badly constructed from the beginning, you cannot fix it later in life. It’s not completely impossible, unlike the genome, which there is limitation you cannot fix it, the epigenome is dynamic.

In this framing, trauma – as a biologically detrimental and impressionable form of stress which acts as a foundation for life trajectories - is literally formative through relatively chronic stimulation of chemical and molecular pathways, especially during early life, and in turn, creates particular kinds of long lasting molecular and cellular change. Trauma is materially unique here in terms of its biological presentation; unlike stress, which is relatively normative, likely to go away in shorter periods of time and even beneficial, trauma represents a significant physiological deviation that, when stable in the sufferer, crystalizes in the form of long-lasting (but not necessarily permanent) biological mechanisms such as epigenetic alterations and memory formation. Similarly, during Alexander’s interview, he was asked the question:

Q: Do you think epigenetics then offers I mean it can act as proof or as evidence the trauma has been there somewhere in the past?

AB: Yes, yes, if it’s done well and yes of course it will show the most proximal mark in DNA, which will show its relationship to stress. And it also provides a psychical mechanism of how stress can be embedded and how stress can be even passed to other generations… So if you were abused as a child it has two components, the abuse itself that happened, that’s history, don’t erase it. But now erase what that abuse *did on your DNA*, because what it did to your DNA is causing you a problem today, so essentially you’re suffering from an abuse that doesn’t exist because it exists in the past.

In this case, traumatic impression – as both epigenetically mediated and as a sign of past events – is characterized not just as a wound inflicted on body, mind or soul, as in the ancient Geek etymology of traûma, “wound, damage”,^4^ but a wound inflicted on DNA itself. And the consequences for neurology and memory are paramount. As we described in the last section, neuroepigenetic researchers postulate that short and long-term memories are in part mediated by epigenetic pathways, and are characterized by the location they are stored in the brain. Not only are differences in location and epigenetic mediation central to the ways in which trauma becomes embedded in the brain, but also to the ways in which it can be “erased” or “treated.” As Lukas explained:

LB: In another study out of my own lab, we showed that long lasting traumatic memories are stored much more differently than recent ones. I mean that short term memories, which we call remote and recent memories respectively, these remote memories, what makes them become stored differently is that their epigenetic make up in their specific brain areas is different, so they are *less plastic*, so they are more condensed and that […] makes them harder to be erased or to be treated with therapy.

Although interviewees pointed out that the evidence supporting neuroepigenetic claims remains thin, and in turn “there is not enough knowledge to know exactly what epigenetic alterations can be repaired,” these statements suggest a kind of speculative future for interventions and treatments whereby micro-matter like epigenetic markers may be targeted; having an epistemological handle on the biophysical and biophysically mediated trace of trauma in the brain raises the possibility of its reversibility, removal and “correction.” In other words, the epigenome, as dynamic and flexible unlike the genome, evokes hope that detrimental consequences of trauma may “not be there forever.”

And yet, conceptually separating the “negative” consequences from the molecular memory of trauma appears to be an unresolved challenge. We could not see passed the politics and ideologies inherent to this kind trauma discourse, as clear in Alexander’s point:

AB: The good news is that if it’s just epigenetic, it could be resolved. There’s evidence from animals that by giving them enriching experiences and so- you know, I think revealing chips on your shoulder is useless. So I don’t get into this, you know, let’s blame past generations for this and for that, that’s useless. I think what we need to learn from history in order to move forward is… and so epigenetics says yes, all the chips are real, but they’re not there forever and with one dramatic change it could disappear…I’m not talking about erasing history, I’m talking about erasing the consequences.

These statements raise a number of significant ethical and conceptual conundrums for researchers in the field, including the ways in which one’s “chips” are judged. Although these seemingly benevolent statements may give credence and “biolegitimacy” (Fassin, 2009) to notions of victimization through embodied trauma, they also draw a sharp and troubling line between “erasable” bodily consequences and external “past” histories. While epigenetics might hold explanatory and even emancipatory power, the pressing question of who, where and what are the violent agents is left wanting.

Attending to the means by which biological (and psychical) processes are relatively malleable, stable, sensitive and responsive opens space for social scientists to think about agents of other kinds, yet it also leaves us wondering how particular modes of violence can be discursively woven into neuroepigenetic analyses when they appear to be exclusively fixed on “purely” biological states. We wonder in particular how much the biological literalism of trauma, where its material truth takes life and validity through imprinted changes or abnormalities in the memory of cells and in the brain, contributes to a dangerous naturalization or reification that divorces trauma “from the complexities of people’s lives and the social structures that give rise to them” (Burstow, 2003). The concern about a losing of complexity has been raised by anthropologists of science, who have pointed to the fact that, for instance in suicide studies, epigenetic notions of trauma are “treated as a black-boxed and dichotomous (i.e., present or not present) category, with the effects of varying experiences in differing contexts generally left undifferentiated” (Lloyd and Raikhel, 2018a,b: 501).

At the same time, we remain open to the idea that neurobiological and new neuroepigenetic evidence may “support ‘what feminists [or other oppressed groups, our note] have known for a long time’ about the effects of trauma” (Tseris, 2013), thus opening up a space for critical dialogue and contestation within biomedical knowledge (Herman, 2015). As Burstow (2003), it makes sense even from a radical stance to keep open a space of critique with “the term and concept [of trauma] nonetheless”: not just because it is advocated by “injured people” or for its cross-cultural resonances but because the phenomenological experience of “soul wound” has a wider circulation particularly in postcolonial contexts (Duran and Duran, 1995; Pihama et al., 2014). More importantly, ceding it to biomedical reductionism is a disempowering gesture from the point of view of the wider social interests and awareness that the work of critique has paved.

Akin to our interlocutors from feminist neuroscience (Roy, 2016; Fine et al., 2017), critical neuroscience (Choudhury and Slaby, 2011) and feminist STS (Haraway, 1988; Wilson, 2004), we believe that an analysis of difference (and sameness) on the scales of neurophysiology and sociality to be a vital progressive step, and one to be made with caution, care and a willingness to experiment with novel methodologies. What remains concerning, however, are the common-sense assumptions in regard sex and gender a number of epigenetic researchers expressed during interviews, which has also been illustrated in feminist STS scholarship (Kenney and Müller, 2017; Richardson, 2017; Saldaña-Tejeda, 2018; Warin and Hammarström, 2018). Importantly, we argue that whilst neuroepigenetic ideations may shed light on the ways in which trauma moves in, through and even out of the brain, we believe that epigenetics as a scientific culture needs to be held accountable for its own conceptual decisions, especially with regard to assertions about gender, race and class. We share the concern of feminist STS that pervasive and concerning notions of gender in epigenetic research need to be addressed, which we explore further.

## Stereotypes and the “Sound of Biology”

In their engagement with epigenetic research, feminist STS scholars have raised concerns about the presence of problematic gendered narratives, tropes and stereotypes, particularly regarding human motherhood. In their article “Of Rats and Women” (Kenney and Müller, 2017: 23) argue that as epigenetic studies “support claims about human motherhood,” they tend to “illustrate rather than interrogate existing stereotypes about maternal agency and responsibility.” Along with Kenney and Müller’s critique, other issues raised by feminist STS include: the exaggerated role of women in transmitting stress to her children and following generations; minimization of other socio-political agents involved in stress inheritance; the questionable use of animal models as a proxy for gauging human behavior; the role of epigeneticists themselves in reinforcing stereotypes of motherhood, particular in public debate (Richardson, 2015); and the potential risk of prospective health policies inherently burdening women with further care responsibilities and adding to already existing surveillance, especially of pregnant women (Richardson et al., 2014). On the one hand, epigenetics and neuroplasticity have been hailed as dissolving boundaries and dualisms, for instance between sex-gender and nature-nurture (Lock and Pálsson, 2016), while on the other, dualistic and essentializing ideas of biological difference remain in the vocabulary and design of epigenetic research.

While the gendered dimension of epigenetic studies has been robustly considered (Kenney and Müller, 2017; Richardson, 2017; Saldaña-Tejeda, 2018; Warin and Hammarström, 2018), and notions of trauma have been analyzed in the context of the epigenetics of suicide risk (Lloyd and Raikhel, 2018a,b), the ways in which gender and sex epistemologically underpin neuroepigenetic studies of trauma is yet to be investigated. To our knowledge, there have not yet been any feminist STS engagements at the intersection of sex, gender, epigenetic imprint and the nervous system; although we do not explore this in great depth here, it is important to highlight that this area requires further attention. Contributing to feminist STS studies on epigenetics, we find that despite the growth of research on paternal epigenetic transmission, the socio-political complexities of parental care and family life in human worlds – when diffracted through a gendered lens – are seldom addressed in neuroepigenetics. Herein, we support the notion that “new stories are old stories” (Kenney and Müller, 2017: 25), and in our case, stories relating to human paternity as well as maternity. Although our aim in this paper is not to offer a resolution or exhaustive critique *per se*, this is an important argument to be making here as we agree that sexism, misogyny and gender-based violence are very much connected to and couched in ambivalent, casual and seemingly harmless beliefs about gender and sex (Vecina and Piñuela, 2017; Testoni et al., 2019).

Many researchers who participated in interviews were - to varying degrees - aware of the issues raised by feminist STS, such as a disproportionate number of maternal studies in epigenetics, and how structural sexism may very well influence research designs, priority areas and funding. However, a substantial number of troubling assertions about sex and gender, as well as nature and nurture, were expressed by interviewees, all of whom were in relatively high positions of authority.

When asked about the disparity between paternal and maternal studies in epigenetics, neuroscientist and epigeneticist Thomas Lai (based at a neuroscience institute in Melbourne) explained:

TL: Historically, medicine has been a very sexist sector, and we are still trying to overcome that. Even nowadays when I give my presentations and I make a passing comment that, hey, the literature is very skewed, we don’t really study paternal effects, I don’t think that that message actually sinks in, or they won’t quite get it.

Lai was critical of the “skewed literature,” yet he explained that there were legitimate reasons, such as: the ambiguous window of time to access sperm RNA prior to conception; technical challenges in breaching the sperm’s casing; as well as difficulties securing the necessary funding for paternal studies. In response to the knowledge gap he saw, Lai has dedicated much of his career to paternal studies:

TL: When it got down to the paternal studies, there was added motivation, because I went, hey, this is – it’s stupid to think that, in terms of pregnancy and infant health, that everything should fall on the woman, that doesn’t make sense at all. So, I thought that needed fixing.

Though for other researchers in epigenetics, the primary focus on “maternal care” in epigenetics is justifiable, given the particular figuration of evolution that is ingrained in human reproduction. During an interview with Alexander Berman, he described parental care-giving as considerably more of a genetic trait in female mammals, less so in males:

AB: [Motherhood] is not something that happened this year right, it’s quite old in evolution and it’s built into the entire system, the way the brain is wired. When an animal becomes a mother, the whole brain is changing. It’s a very strong thing. Humans learn to be fathers but it’s more learned, it’s less evolutionarily engrained, that’s why you still have problems. So with all the politics and philosophy and changes in attitudes still […] overall fathers would probably disappear more than mothers, and so you can take an animal model so far but in the end, I think evolution takes over and this is a big lesson.

A similar point was raised both by Adam Weber, a neuroscientist working on paternal epigenetic studies (Melbourne), and Connor Ringwood, who works in epigenetic tagging and brain development (Perth). For Adam, mothers are realistically the most influential on their offspring given their role in gestation, birthing and breastfeeding:

AW: I wouldn’t say there’s an over-representation [of maternal studies], because the reality is, biologically the father. you know, passes on this genetic, and we think, epigenetic information at conception, and then all of the influence is pretty much with the mother, what she does during pregnancy, what she does while she’s breastfeeding, early maternal care, obviously postnatally the father comes into play more, in mammals anyway, in some mammals like humans.

For others like Connor, given the particular reproductive role of mothers it makes sense that there is understandably more responsibility placed on them:

CR: Well at the end of the day there has to be, there is naturally going to be more responsibility placed on the mother because they carry the next generations within them, whether blame should be exercised is a different matter, but if it’s found that, we know there are behaviors that can have profound effects on the next generation, alcohol consumption for example, when that link is clearly made I think people then. it’s much easier to rationalize and justify why certain behaviors need to change.

A particular figuration of human reproduction is inherent to these comments from Alexander, Adam and Conner; on the one hand, a mother’s biology (in other words, her nature) readily changes in the process of childbearing and rearing, while fathers tend to naturally *stray*. And importantly, as Alexander went on to emphasize, these behavioral signs of evolution are, by virtue of their genetic imprint, slow to change. Yet the other hand, as he says, there is an urgency to “listen” to the sounds of evolution and biology:

AB: Whatever political ideas we have, we do have to be attentive to the sounds of evolution and biology, right? There are some things that are going to be really hard to change if they’re so deeply wired in our genome. This is not epigenetic, this is genetic, this is evolution.

A tension arises between what constitutes as “epigenetic” and “genetic” here, or to put in another way, between modes of evolutionary adaption inherent to human reproduction. While genetic and epigenetic dispositions here become seemingly synonymous with evolutionary accounts of nature and nurture respectively, we can’t help but notice the unsettling authority of gene-centric doctrine raising its head (Haraway, 1976; Keller, 1997, 2002). What we find especially troubling in these framings is the kind of erasure they have the potential to cause. There is violence inherent to stereotyping (Dobash and Dobash, 1992; Butler, 1999; Gilbert, 2002) as much as there is potential harm inherent to trauma conceptions that downplay or are inadequately informed by knowledge of violence. In the somewhat idealistic representations of gender and gender roles illustrated above, there is a risk not only of lived and gendered experience falling out of the analytical picture, but of the finer “specificities of care” failing to receive the empirical attention they need (Mol et al., 2010: 9).

Drawing from the quotes above, they illustrate a need for biological scientists, and especially those in leadership positions, to be mindful of rehashing dogmatic evolutionary ideas about reproduction, familial care and gender roles. If neuroepigenetics provides a mouthpiece for neurobiology, and if biology’s “voice” is akin to the sound of lived experience, might this not sound like gender politics, indeed, sound like politics in general? Rather than making old stories new stories, an attentiveness to the sounds of biology may in fact steer us toward legacies of embodied experience, as well as other agents and other ways of listening. Clearly, the issue of where and who agents are calls for cross-disciplinary dialogue, as it makes little sense to qualify a reactive genome and biologized environment when the epigenetic narrative reduces its own claims to questions of (mal)adaptive mothering and reason-able fathering. In the final section of this paper, we discuss some of the reasons for why collaboration is crucial now, and why social and biological scientists need to be talking to each other about matters of gender, trauma and embodiment.

## There is *So* Much Going on in Real Life: Collaborative Research Directions

After a number of attempts to strike a suitable time, one of us (EL-B) sat to a Zoom interview with Ian Tremblay, an epigeneticist and molecular biologist based in Melbourne. Before proceeding with the interview, Ian talked compassionately about some of the pandemic’s many indirect victims: small business owners, international students, hospitality workers, elderly folk, and in particular, those surviving family violence. The daily news had rendered Ian melancholy, as he said:

IT: I looked up a word “weltschmerz,” it means “world-pain.” It’s kind of like, when you feel for the whole world… I think it affects everybody, there are layers of a dark heavy blanket over everyone.

In light of restrictions, curfews, isolation, financial hardship and other factors that have worsened in response to the pandemic, advocates have raised concerns about a rise in family violence across the globe. As trauma psychologists [full name] (Kofman and Garfin, 2020: S199) have said:

The novel coronavirus (SARS-CoV-2) and the associated disease it causes, COVID-19, have caused unprecedented social disruption. Due to sweeping stay-at-home orders across the United States and internationally, many victims and survivors of domestic violence (DV), now forced to be isolated with their abusers, run the risk of new or escalating violence.

Like other enduring and complex social issues, family violence has acquired a face in the pandemic. Yet, at least in Australia, statistics from the last ten years have been described as indicating a “national crisis” and “silent epidemic,” with a third of women experiencing physical violence in their lifetime (Piper and Stevenson, 2019). Throughout the COVID-19 pandemic an array of social injustices have come to the forefront: low welfare payments, precarious casual workforces, racialized inequality and especially, the rates of family violence which overwhelmingly victimize women (Kofman and Garfin, 2020; Mazza et al., 2020; Fullagar and Pavlidis, 2021). We have argued in this paper that neuroepigenetics holds opportunity for creatively thinking about gender and gender-based harm as embodied (Blackman, 2011, 2016; Niewohner, 2015), yet the ways in which family and intimate violence eventuates at global, local and temporal scales pulls us into difficult interdisciplinary territory and exposes a rift between biological and social epistemologies.

During interviews, researchers themselves indicated a pervasive and at times frustrating issue with visibility (i.e., what can be seen) and invisibility (what remains hidden), and thus we do not wish to add unnecessary – and unhelpful – fuel to the fire. Yet, when neuroplasticity assumes environments, traumas and – in our case, genders – to be absorbable, the question presents itself of how we can hybridize and harmonize different methodologies in order to engage bodies, brains, modes of care and environments, without losing the finer nuances of these terms. As (Blackman, 2016: 269):

Epigenetics holds promise to qualify the relations between biology, psyche and trauma, yet paradoxically, it is also at risk of erasing the nuances of lived, embodied experience in its attempt to discursively molecularise the environment.

This point was especially salient in regard to one particular interview with neuroscientist Sasha Reed (Melbourne). At the time of our interview, Sasha was working with Serbian perinatal women who had experienced sexual trauma throughout the wars in Kosovo, a study which exemplified the trauma socio-political contexts can bring - quite literally - into life.

As Sasha explained, the aim of her project was to examine how epigenetic changes responsive to trauma may be passed onto the women’s children. Though Sasha’s research team were also interested in the kinds of supportive interventions available to survivors, such as social support and counseling, and how these may influence epigenetic modification. Sasha noted that many of the women using these services were doing so without their husband’s knowledge; a reason being that sexual trauma was not to be talked about within their family unit. Unlike animal studies which are relatively much easier to control, this kind of local and human-based research proved challenging for Sasha’s team; not only due to the substantially complex amount of agential factors requiring their consideration, but also because of the lack of control they had over the variables. This left Sasha feeling bereft, as she explained:

SR: We are trying to do this kind of intergenerational research, but it’s tricky I mean. It’s very tricky. Just because there’s so many other factors that you know you can’t control. It’s not an experiment. It’s real life.

If there’s one important point to be gained from Sasha’s statement, it would help to return to a point we raised in this paper’s introduction. In some ways, a feminist ethos has been innate to epigenetic research because of its decentering of DNA and heightened attention to contexts both internal and external to a cell’s nucleus (Keller, 1988, 2002; Haraway, 2007; Malabou, 2010). In the example of Sasha’s human-based research, the ways in which gender is lived, experienced, embodied and even inherited insists on epistemological and methodological attention. The team’s position in relation to their research subjects matters, and we assume, to the degree of making methodological trouble. There is *so much* going on in “real life” – which we find to be a sobering realization that unsettles any clear divide between nature, nurture, biology and culture. Importantly, we believe that “real life” insists on taking pause to hear from and know about the people whose neuro-social narratives are being written, as well as how specific forms of violence may be illuminated or obscured by epistemological processes.

Perhaps a feminist ethos in this context – and indeed a feminist neuroepigenetics – could be to befriend ambiguity and use it as a touchstone for cross-disciplinary orientation. As hidden forms of violence persist – attached or not to civil war, famine or political unrest – neuroepigenetic methods are left wanting of other perspectives, and researchers themselves are pointing this out. Indeed, in many instances their accounts echoed the classic feminist catch phrase “the personal is political”; during an interview, one epigenetic researcher talked about an article he recently read about abusive relationships:

IT: [It] was talking about these kinds of relationships, dependent relationships. I spoke to [the author] afterward because my niece-in-law is in one of those relationships with someone who’s violent. And they keep going back, it seems to be one of those stories doesn’t it, where it’s, it’s a common issue where it’s some dependency. It’s like the two people seem to go together, the abuser and the abused. It’s not the fault of the abused, but it’s just a known dynamic isn’t it, I think, and you really feel, externally you – people go, why do you go back to that person? But it just, it just happens doesn’t it?

Feminist STS theorist Donna Haraway (1988) writes that in knowledge-making endeavors, foregrounding social, political and environmental issues raises the risk of backgrounding others. When reflecting on “the layers of a dark heavy blanket” Ian described, it raises concern about the invisibility, complexity and multiple scales of gender-based violence, and how such harms endure whether or not epistemological tools allow access. Historical epistemology makes us aware of the malleability of concepts and scientific tropes, as well as their open-ended political nature: even if rooted in a gendered imagination (for instance the figure of the hysterical woman in the nineteenth and early twentieth century: see Gilman et al., 1993; McDonald, 2018) or in premodern thought (the patriarchal assumption of female passivity in embryological processes on which marks can be left by the active power of the male semen), there is opportunity to model for wider processes of inscription and even trauma on memory (Lurz, 2008; Meloni, 2019). Feminist appropriations of trauma have occurred and are certainly possible (Brown, 2004) and similar work can be possibly done for its present neuroepigenetic iteration.

Although our goal here is not to provide a succinct roadmap for the kinds of collaborative research we hope will eventuate in this space, one practical suggestion we can make is a basic one: improve and increase communication. We say this because the process of interviewing epigenetic researchers was not a straightforward one. It took time for people to make the time to talk; it took them having time in the first place; and once the practicalities of working out time-zones and Zoom invitations were sorted, it took time to find common ground while mutually understanding the different methodologies and worldviews we have. It took many attempts to get the words right. Yet, we found that the most productive conversations were the most basic, honest and mutually understandable. They yielded rich dialogue, and moreover, a foundation to build “critical friendships” upon (Rose and Abi-Rached, 2013; Fitzgerald and Callard, 2015), which we are sure will prove to be fruitful and sustainable.

## Conclusion

If epigenetics is indeed an opportunity to pinpoint moments where experience and biology meet, a useful question to ask, then, might be: if representations of biology, biological change and responsiveness can ethically account for environments, histories, politics and adversities, who and where are the agents we account for? As feminist STS scholars and medical anthropologists have shown, there is a necessity to turn toward a multiplicity of narratives, story and truth telling. This may help us to better grasp not a mono-objectivity of biology, but an objectivity that accounts for many perspectives (Haraway, 1988). In this paper, we have illustrated how epigenetics does indeed stand in complex relation to sex and gender, especially with regard to the kind of figurations of reproduction and inheritance. Yet we also find that neuroepigenetics offers a bounty of opportunity not only to consider biological and neurological agents in figurations of embodied trauma, but also to conceptualize gender as biologized. In regard to the prospect of collaborative research, our aim beyond this article is to foster cross-disciplinary discussions and gather together a plurality of voices, for instance by beginning with a neuroepigenetics and trauma online symposium. A useful model for this format is based on recent biosocial initiatives like the 2019 “Symposium on Biosocial Approach to Population Health Across the Life Course,” hosted by the Carolina Population Center (CPC). The aim of this symposium was to:

stimulate novel opportunities for biosocial health research by developing a scientific forum that provides emerging scholars a chance to present research, while facilitating the integration of social and biological approaches for addressing the complex health concerns of today^5^.

Similarly, through a cross-disciplinary symposium that brings neuroepigenetic, feminist STS and medical anthropology into conversation, we see opportunity for novel biosocial approaches to trauma to be generated. We anticipate that the challenge of communication will surface when members of different camps arrive at the table. Thus, we suggest that the act of listening and respecting knowledge others arrive with to be essential. We understand some scientists do not want to enter into the gender-sex debate, or deal with so-called “semantics.” If we may respond, we would say that unfortunately, this is not a chance we are given: for better or worse, all parties already have. For its entangled biosocial nature, epigenetics *does have* political traction and so each epistemological claim will be served best with cross-disciplinary discussion and accountability. We arrived at this project as students of epigenetic knowledge and have learned that trauma has no single ontology, even or perhaps above all in a standardized lab setting. One role of interdisciplinary discussion then is to unpack and unpick assumptions about gender in epigenetics, and to build generative methodologies that can engage with a plethora of agents.

## Data Availability Statement

The raw data supporting the conclusions of this article will be made available by the authors, without undue reservation.

## Ethics Statement

The studies involving human participants were reviewed and approved by the Human Ethics Advisory Group (HEAG) – Deakin University. The participants provided their written informed consent to participate in this study. All identifying markers have been replaced with pseudonyms.

## Author Contributions

EL-B led the drafting of the article, and contributed excerpts from qualitative interviews conducted as part of her Ph.D. MM contributed a wealth of citations, material on the history and conceptualization of trauma, editorial suggestions, and provided support for the article’s aims and focus. Both authors contributed to the article and approved the submitted version.

## Conflict of Interest

The authors declare that the research was conducted in the absence of any commercial or financial relationships that could be construed as a potential conflict of interest.

## Footnotes

1 Interviews were conducted internationally by one of us (EL-B) over the course of 2020 as part of a Ph.D. project at Deakin University, *Biological biographies and molecular memories: A study of epigenetics and how trauma gets under the skull*.

2 In adherence with this project’s ethical parameters, all identifying markers in transcripts have been removed and replaced with pseudonyms.

3 https://www.sciencedaily.com/releases/2015/01/150121114604.htm

4 As Leys notices, “Trauma was originally the term for a surgical wound, conceived on the model of a rupture of the skin or protective envelope of the body resulting in a catastrophic global reaction in the entire organism. Yet as Laplanche has emphasized, it is not easy to retrace the “transposition” of this medicosurgical notion into psychology and psychiatry.” (2000:19)

5 https://cpha.duke.edu/news-events/news/dupri-participates-cpc-symposium-biosocial-approaches-population-across-life
